# Hospital and Institutional News

**Published:** 1917-04-07

**Authors:** 


### HOSPITAL AND INSTITUTIONAL NEWS.

### THE POSTMASTER-GENERAL AND THE SATURDAY FUND.

It is easy to nag at Government Departments, and criticism of public bodies is a thing to be welcomed in itself; but few people will not share the feeling of dismay produced by the action of the Post Office in regard to its recent treatment of the Hospital Saturday Fund. When the penny-a-week collecting sheets for the past quarter were dispatched by the Saturday Fund's officers to the W.C. District Post Office in December last, the overseer decreed that these sheets could only be transmitted at the letter rate, and no longer be regarded, and charged for, as printed matter. The cost of their posting was therefore at once trebled, and rose from £6 5s. to £19. A protest being made, the Postmaster-General's secretary fortified himself by the technical objection that there was no provision in the regulations for the transmission of these blank articles of stationery at the Inland Revenue postage rate. Since economy of management is the necessary order of the day, the executive of the Fund is seeking to deliver the collecting sheets by hand, or other means independent of the Post Office, and we support the Fund's plea that "some friend of the Metropolitan hospitals should take the matter up" in both Houses of Parliament. An official often tends to be penny wise and pound foolish, but it is well to remind the official who loves the letter of his law that the present is hardly the time to hamper the work of the hospitals, who are loyally carrying a heavy burden in the war; and, moreover, that even law itself recognises the force of prescriptive right, a right which the Hospital Saturday Fund has undeniably acquired in the present matter.

### UNIVERSITY COLLEGE HOSPITAL MEETING.

A remarkable development of work has taken place at University College Hospital, which shows the demand made by the war. In the past official year the number of in-patients increased by 832 over the previous year's figures, and to provide the necessary accommodation the number of beds had been increased to 492, which, be it noted, have been evenly distributed between civil and military cases. The wounded sailors and soldiers treated in the military wards numbered 1,705, the Government grant for whom amounted to £13,237. The Tuberculosis Department is now established as a treatment centre for Holborn and St. Pancras, under the Metropolitan scheme, and the work of the Maternity and Child-Welfare Department has been much extended and two observation cots have been installed in the children's ward. In spite of a fall in the sum derived from legacies, the ordinary income amounted to £39,981, which was very little short of the total expenditure. Mr. J. Gerald Buckle, the secretary, has received special thanks for his work at this difficult time. An attempt is being made to increase the number of cots, and beds have been already installed for the treatment of cases of venereal disease. This is a fine record, and the expansion of the hospital shows that the public conscience has only to set the pace for the voluntary hospitals to follow. The hospital had maintained in every way a high standard in regard to medical and surgical requirements. He hoped the public would realise that the hospital required continued support in order to keep it up to the present high standard. The report was adopted.

### SOME GENEROUS BENEFACTIONS OF THE WEEK.

Once again, as so often in the last few weeks, the voluntary hospitals have had a good run of legacies. The largest individual legacy is that of £10,000, bequeathed by Mr. Thomas Hellyar Foord to St. Bartholomew's Hospital, Rochester, for which institution this gentleman had already during his lifetime built and furnished a nurses' home at a cost of £6,000. The residuary estate, which will reach a substantial sum, is left for the building of almshouses at Rochester. Mr. Foord was ninety-three years of age, and another nonagenarian benefactor of the week is Mr. Francis Reckitt (of Reckitt's Blue fame), who died a millionaire, and has left £5,000 each to the Great Northern Central Hospital for their nurses' home and to Dr. Barnardo's Homes. He also devised £2,000 to King Edward's Hospital Fund, and £1,000 each to a number of charities, including the Royal Hospital for Incurables, Putney; the Mount Vernon Hospital for Consumption and Diseases of the Chest; the Hull Royal Infirmary; and the Hull and East Riding Convalescent Home. The Hull Children's Hospital and the Cancer Hospital, Brompton, obtain £500 each under the same will. Then we have the will of Miss Caroline Emma Chretien, of Bexley Heath, who has left the proceeds of the sale of a house to the Bexley Cottage Hospital, to endow a "Chretien Wing or Ward." Of the balance of her real estate, one-third is left to the Poplar Hospital for Accidents. To the London Hospital she bequeathed various "gilt-edged" securities of the total nominal value of nearly £7,500, though the actual value, owing to depreciation, is a good deal less than this.

### THE PAY OF SUPERINTENDENTS OF CIVIL WAR HOSPITALS.

A recent decision of the War Office concerning the pay of the superintendents of war hospitals established in Poor-Law buildings is causing a good deal of anxiety to the medical men concerned, as well as to the Boards of Guardians who employ them. The facts would appear to be that when these infirmaries were turned into war hospitals the superintendents were given commissioned Army rank (as lieutenant-colonels or as majors), with an understanding as to remuneration which is now to be upset. The understanding was that each superintendent should have either (a) his normal salary

Page(s) missing hospital at Klyster. By bathing all the sufferers—even when yelling in delirium—every day in baths containing crude carbolic and naphtha soap, the scabbing was hastened, the markings reduced, and the recovery rate greatly improved. Dr. Atkinson made a strong appeal for help for the units, the treasurer of which is Mrs. Chapman, Burnage Lodge, Levenshulme.

### LOOKING AHEAD TO A PENSION.

Most hospital secretaries are aware that from time to time their boards of management expect them to suggest the names of likely gentlemen for election to the governing body. It is the custom of some secretaries to nominate elderly men who are known to be well-to-do and "sympathetic." No doubt the secretary imagines that the elderly and well-to-do man is likely to prove clay in the hands of the potter! But surely this is very short-sighted policy, because the hospital secretary runs the risk of outliving his friends and finding himself comparatively unknown to his board at the critical time when his pension comes to be considered. With this in mind, one hospital secretary we know has successfully suggested several men to his own board, and their ages have been much about the same as his own. But the idea is not altogether selfish, for services are enlisted of men who are in the prime of life and whose business aptitude is likely to be of greater service than that of men who are about to reach the allotted span of life. Happily the present haphazard and unfair method of awarding pensions to hospital officers is likely soon to become a thing of the past as regards London hospitals, for King Edward's Hospital Fund have under consideration a scheme to place the whole matter on an equitable basis.

### A RECORD OF SOLDIERS' SONGS.

A word is due to a recent number of the *Searchlight* for the article contained therein entitled "Some Soldier Songs and their Singers." It gives excerpts from some of the most popular, and, as a result of his researches, the writer declares that "the most popular are those which mention the soldier's solace—beer." It may well be so, especially when he explains that it is the "trickle of words" as much as the beverage itself which makes these songs so popular. This would please Mr. Chesterton, who once wrote a fine drinking-song about the flood, which had for its refrain this exclamation of Noah: "I don't mind where the water goes, as long as it does not get into the wine." We are glad to hear that this article on the subject of soldiers' folk-lore is to be continued. The emendations are often admirable; why not an anthology of the best?

### A RECURRING DIFFICULTY FOR HOSPITAL SECRETARIES.

Bishop Welldon, the present Dean of Manchester, said recently: "Three days ago I had a postcard from Bath in a handwriting which I take to be that of a maiden lady, saying that I am an old ostrich." The Dean must be a particularly discerning man, for most hospital secretaries are bewildered daily by handwriting from ladies who expect a letter in return and who do not say whether they are a "Mrs." or a "Miss." It would be gratifying to learn from the Dean how he discriminates between the writing of a married and an unmarried lady. When a hospital secretary addresses a married lady as "Miss" he runs the risk of forfeiting all her future interest in his institution! Deans do not live by "collections," but most hospital secretaries do; so that, in any case, the Dean has less to fear from a mistake with regard to handwriting than has a hospital secretary. No doubt when Bishop Welldon was headmaster of Harrow School he received more letters from anxious mothers than he did from fussy maiden aunts, and this experience may have proved a useful guide to him in his calligraphic investigations.

### NO DISCRETION FOR NURSES.

Nurses are not allowed to exercise discretion. The Local Government Board transmitted to the Lambeth Board of Guardians a communication received from Canon W. E. Hague, of St. Paul's Lodge, Clifton, Bristol, complaining that on the removal of a man, for admission to the infirmary recently, he was stripped of all clothing and wrapped in a blanket. It is suggested that as the man in question was very respectable such extreme measures were not justified. In a statement submitted to the Guardians, the medical officer of the infirmary stated that, in his opinion, it is most undesirable that the nurse responsible for the removal of patients to the infirmary should use any discretion as to whether or not patients should be removed in their own clothes or in blankets, and that if a patient is properly handled he cannot see that the latter course, which is adopted in every case, should give any offence, as the patient is completely wrapped in the blanket, which is pinned so as to avoid any exposure.

### THIS WEEK'S DRUG MARKET.

The drug market is naturally quiet owing to the near approach of the Easter holidays, but the upward movement of prices is still very pronounced. Strychnine is again dearer. The hope that the value of atropine would again be reduced has been disappointed, for the price is higher again; homatropine is also rather dearer. Balsam copaiba has become scarcer, and prices are again higher. Stocks of cascara sagrada are low, and the price is well maintained. Myrrh is very scarce. Honey still tends upwards in price. Very little henbane extract is offered. Best qualities of olive oil are difficult to obtain. Opium still tends upwards in price. Hydrastis canadensis is dearer. It is not altogether improbable that prices of bromides will be advanced. In synthetic drugs there is very little change, and nothing has occurred to relieve the scarcity of barbitone. One of the very few drugs for which lower prices are quoted is lanoline, which is now being produced in this country in good commercial quantities.

